# Genome Sequence of the Small-Colony Variant *Pseudomonas aeruginosa* MH27, Isolated from a Chronic Urethral Catheter Infection

**DOI:** 10.1128/genomeA.01174-13

**Published:** 2014-01-23

**Authors:** Petra Tielen, Daniel Wibberg, Jochen Blom, Nathalie Rosin, Ann-Kathrin Meyer, Boyke Bunk, Max Schobert, Reinhilde Tüpker, Sarah Schatschneider, Christian Rückert, Andreas Albersmeier, Alexander Goesmann, Frank-Jörg Vorhölter, Dieter Jahn, Alfred Pühler

**Affiliations:** a Institute of Microbiology, Technische Universität Braunschweig, Braunschweig, Germany; b Center for Biotechnology, CeBiTec, Universität Bielefeld, Bielefeld, Germany

## Abstract

*Pseudomonas aeruginosa* is a notable nosocomial pathogen causing severe chronic infections. Here we present the draft genome sequence of *P. aeruginosa* MH27, isolated from a patient with a chronic hospital-acquired catheter-associated urinary tract infection. The 7.1-Mb genome sequence organized in 24 scaffolds contributes to the understanding of biofilm formation and antibiotic resistance.

## GENOME ANNOUNCEMENT

*Pseudomonas aeruginosa* is one of the most frequent pathogens causing complicated catheter-associated urinary tract infections (CAUTIs) (1, 2). Such infections are based on the formation of biofilms (3). Highly adherent small-colony variants of *P. aeruginosa* reveal enhanced ability to form biofilms (4) and express resistance to antibiotics (5). These are major problems during eradication of these infections, which may become chronic as a consequence.

Here we describe the draft genome of *P. aeruginosa* MH27 isolated from a chronic CAUTI. The strain exhibits a small-colony morphology typically known from isolates of chronic cystic fibrosis lung infections (5) resulting in a highly aggregative phenotype. Moreover, the strain is cytotoxic and multidrug resistant and exhibits high production of virulence factors (6, 7).

To obtain the draft genome sequence, we extracted genomic DNA of *P. aeruginosa* MH27 to construct a paired-end library for shotgun sequencing with the Genome Sequencer FLX (GS FLX) system using Titanium technology (Roche) as described recently (8). Standard protocols were followed according to the manufacturers' instructions. Assembly with the GS De Novo Assembly software (Newbler) covered 228,274,133 bases from 1,035,681 aligned individual reads, among them 228,178 paired-end reads. The assembly resulted in 31 contigs that were organized into 24 scaffolds by using the paired-end information. The scaffolds covered 7,155,691 bp, with an average coverage of 32× by shotgun reads. The genome had a G+C content of 65.87%.

Automated genome annotation was carried out using the GenDB software (9). After automated prediction and subsequent manual curation, 6,297 protein-coding sequences (CDS) and 78 RNA-coding genes were identified for the *P. aeruginosa* MH27 genome. Three copies of the 5S, 16S, and 23S rRNA genes were identified, and 63 tRNAs were predicted. The genome sequence was compared with the core genome of *P. aeruginosa* using the software EDGAR (10). Thereby, 226 unique CDS were identified in the MH27 genome. Among these, 39 genes were found to be involved in metabolism. We concluded that the metabolism is specialized for adaptation to urinary tract conditions. Furthermore, several genes were found that are involved in osmotic stress protection, like the Na^+^/H^+^ antiporter gene *nhaA* (PAMH27_2188) and a gene encoding a compatible solute glycine betaine transporter (PAMH27_5169 to PAMH27_5171). Enhanced antibiotic resistance was correlated to genes coding for the mercuric reductase MerA (PAMH27_5101), TniB (PAMH27_6063), which is involved in mercury resistance and transposition, and a transcriptional regulator of the AmpR family (PAMH27_5166) and to genes for the efflux pump OpmR (PAMH27_0614) and for a drug resistance-mediating transporter (PAMH27_5898 to PAMH27_5890).

Functional analyses based on comparative genomics utilizing this genome will facilitate a deeper understanding of urinary tract infections caused by *P. aeruginosa*.

### Nucleotide sequence accession numbers.

Sequence data related to this whole genome shotgun project have been deposited in DDBJ/EMBL/GenBank under the accession no. CBTR000000000. The version described in this paper is the first version, CBTR000000000.1.
